# Supplementary material for: Joint influences of obesity, diabetes, and hypertension on indices of ventricular remodeling: Findings from the community-based Framingham Heart Study
Source: PLoS One. 2020 Dec 10;15(12):e0243199. doi: 10.1371/journal.pone.0243199 (PMC7728232; doi:10.1371/journal.pone.0243199)
Supplement: S1 Fig — Abbreviations: CHF: congestive heart failure; Gen 3: Third Generation Framingham Cohort; Offspring: Framingham Offspring Study; Omni 2: Omni 2 Cohort. (DOCX) [file pone.0243199.s001.docx]

**
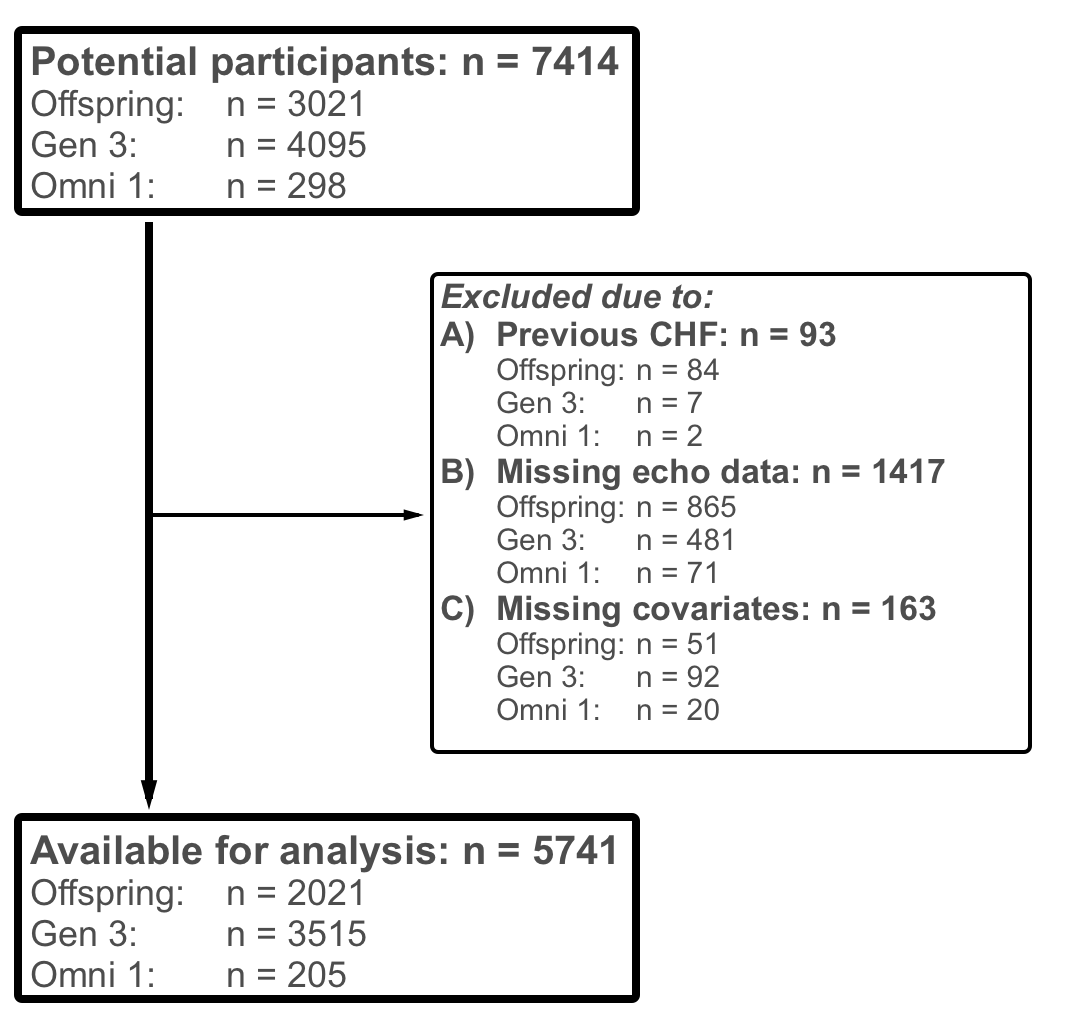
**

**S1 Fig.** Flow diagram representing selection and exclusion process. Abbreviations: CHF: congestive heart failure; Gen 3: Third Generation Framingham Cohort; Offspring: Framingham Offspring Study; Omni 2: Omni 2 Cohort.
