# Supplementary material for: Joint influences of obesity, diabetes, and hypertension on indices of ventricular remodeling: Findings from the community-based Framingham Heart Study
Source: PLoS One. 2020 Dec 10;15(12):e0243199. doi: 10.1371/journal.pone.0243199 (PMC7728232; doi:10.1371/journal.pone.0243199)
Supplement: S2 Fig — Least squares means of left ventricular mass indexed by height (A), left ventricular mass indexed by body surface area (B), left ventricular wall thickness (C), relative wall thickness (D), mitral annular plane systolic excursion (E), and negative global circumferential strain (F) according to cross-classified body mass index (normal weight: BMI < 25kg/m2, overweight: 25kg/m2 ≤ BMI < 30kg/m2, obese: BMI ≥ 30kg/m2)) hypertension, and diabetes status categories. All models are adjusted for cohort, age and sex. P values correspond to 3-way interactions. (DOCX) [file pone.0243199.s002.docx]

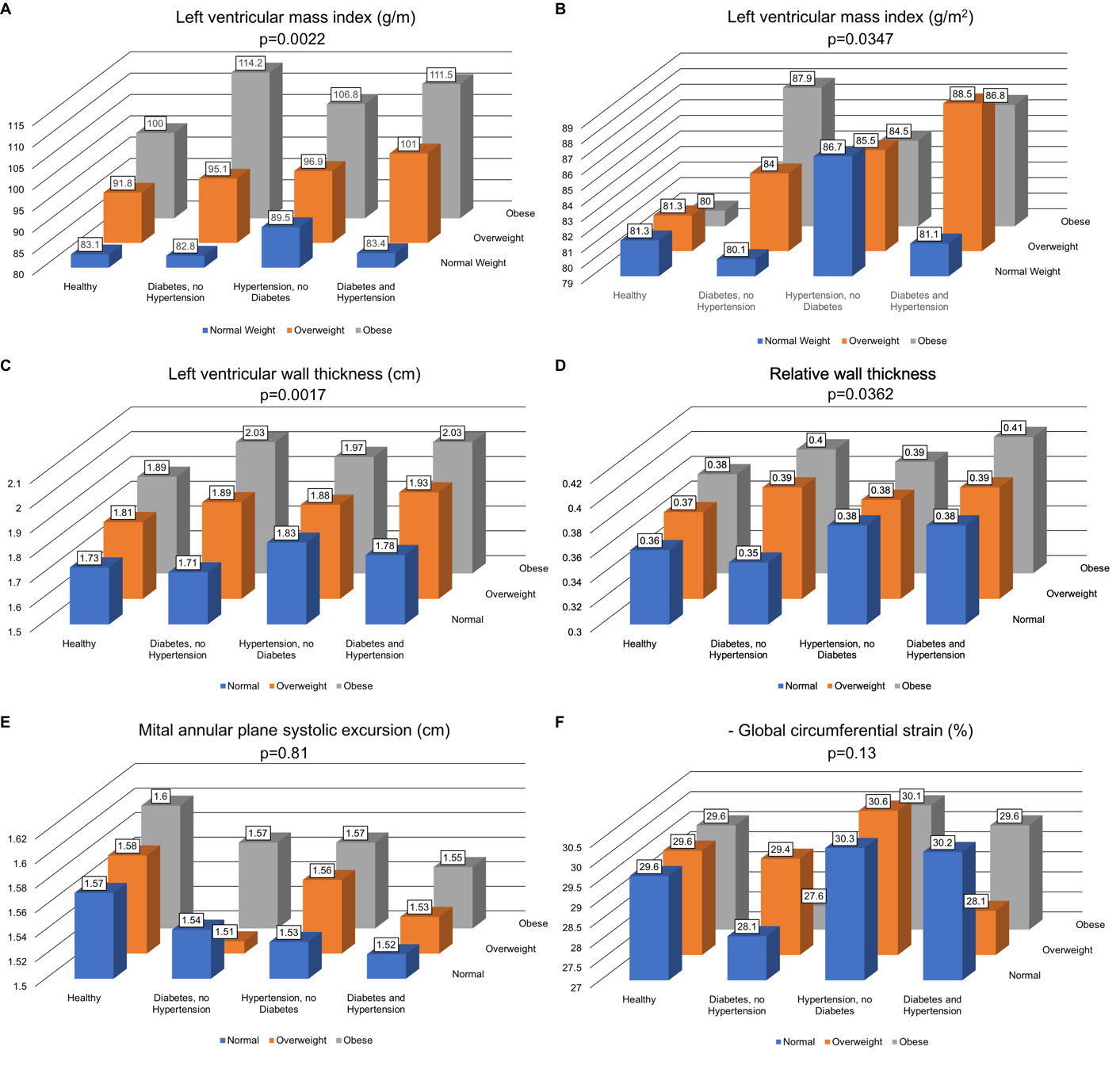


**S2 Fig.** Least squares means of left ventricular mass indexed by height (A), left ventricular mass indexed by body surface area (B), left ventricular wall thickness (C), relative wall thickness (D), mitral annular plane systolic excursion (E), and negative global circumferential strain (F) according to cross-classified body mass index (normal weight: BMI < 25kg/m^2^, overweight: 25kg/m^2^ ≤ BMI < 30kg/m^2^, obese: BMI ≥ 30kg/m^2)^) hypertension, and diabetes status categories. All models are adjusted for cohort, age and sex. P values correspond to 3-way interactions
