# Supplementary material for: Joint influences of obesity, diabetes, and hypertension on indices of ventricular remodeling: Findings from the community-based Framingham Heart Study
Source: PLoS One. 2020 Dec 10;15(12):e0243199. doi: 10.1371/journal.pone.0243199 (PMC7728232; doi:10.1371/journal.pone.0243199)
Supplement: S1 Table — Least squares means of left ventricular mass index (LVMI) indexed by height (g/m), height^2.7, and body surface area (g/m2), left ventricular wall thickness (LVWT), relative wall thickness (RWT), mitral annular plane systolic excursion (MAPSE), global circumferential strain (GCS) according to body mass index (BMI) category (normal weight: BMI < 25kg/m2, overweight: 25kg/m2 ≤ BMI < 30kg/m2, obese: BMI ≥ 30kg/m2), hypertension status (HTN), and diabetes status (DM). All models are adjusted for cohort, age, sex. Bold print: p < 0.05, considered significant in secondary analysis. (DOCX) [file pone.0243199.s003.docx]

**S1 Table.** Least square means of echocardiographic traits by BMI category**,** hypertension status and diabetes status (modeled separately) in secondary analysis, adjusted for age, sex, and cohort.

| **Parameter** | **BMI Category** | | | | **Hypertension Status** | | | **Diabetes Status** | | |
| --- | --- | --- | --- | --- | --- | --- | --- | --- | --- | --- |
|  | Normal Weight  (39%) | Over-weight  (38%) | Obese  (23%) | P Value | No HTN  (70%) | HTN  (30%) | P Value | No DM  (95%) | DM  (5%) | P Value |
| **LVMI-height, g/m** | 84.3 | 93.5 | 103.7 | **<0.0001** | 89.3 | 98.6 | **<0.0001** | 91.5 | 100.8 | **<0.0001** |
| **LVMI-height^2.7, g/m** | 34.5 | 38.4 | 42.7 | **<0.0001** | 36.6 | 40.7 | **<0.0001** | 37.6 | 41.4 | **<0.0001** |
| **LVMI-BSA, g/m^2^** | 82.4 | 82.7 | 82.4 | 0.71 | 81.2 | 85.5 | **<0.0001** | 82.3 | 85.4 | **0.0004** |
| **LVWT, cm** | 1.75 | 1.84 | 1.94 | **<0.0001** | 1.79 | 1.90 | **<0.0001** | 1.82 | 1.92 | **<0.0001** |
| **RWT** | 0.37 | 0.37 | 0.39 | **<0.0001** | 0.37 | 0.39 | **<0.0001** | 0.37 | 0.39 | **<0.0001** |
| **MAPSE, cm** | 1.56 | 1.57 | 1.58 | **0.02** | 1.58 | 1.56 | **0.004** | 1.57 | 1.55 | **0.046** |
| **GCS, %** | -29.7 | -29.9 | -29.7 | 0.51 | -29.6 | -30.2 | **<0.0001** | -29.8 | -28.8 | **0.0003** |

Least squares means of left ventricular mass index (LVMI) indexed by height (g/m), height^2.7, and body surface area (g/m^2^), left ventricular wall thickness (LVWT), relative wall thickness (RWT), mitral annular plane systolic excursion (MAPSE), global circumferential strain (GCS) according to body mass index (BMI) category (normal weight: BMI < 25kg/m^2^, overweight: 25kg/m^2^ ≤ BMI < 30kg/m^2^, obese: BMI ≥ 30kg/m^2^), hypertension status (HTN), and diabetes status (DM). All models are adjusted for cohort, age, sex.

Bold print: p < 0.05, considered significant in secondary analysis
