# Supplementary material for: Joint influences of obesity, diabetes, and hypertension on indices of ventricular remodeling: Findings from the community-based Framingham Heart Study
Source: PLoS One. 2020 Dec 10;15(12):e0243199. doi: 10.1371/journal.pone.0243199 (PMC7728232; doi:10.1371/journal.pone.0243199)
Supplement: S2 Table — Least squares means of left ventricular mass index (LVMI) indexed by height, height^2,7, and body surface area, left ventricular wall thickness (LVWT), relative wall thickness (RWT), mitral annular plane systolic excursion (MAPSE), and global circumferential strain (GCS) according to body mass index (BMI) category (normal weight: BMI < 25kg/m2, overweight: 25kg/m2 ≤ BMI < 30kg/m2, obese: BMI ≥ 30kg/m2), hypertension status (HTN), and diabetes status (DM). All models are adjusted for cohort, age, sex. P-Value <0.05 was considered significant for interaction terms. (DOCX) [file pone.0243199.s004.docx]

**S2 Table.** Least square means of echocardiographic parameters stratified by BMI category, hypertension status and diabetes status (modeled jointly) in secondary analysis, adjusted for age, sex, and cohort.

| **Echo Parameter** | **BMI Category** | **Healthy** | **DM, no HTN** | **HTN, no DM** | **HTN and DM** | **P-Value for 3-way interaction** |
| --- | --- | --- | --- | --- | --- | --- |
| **LVMI-height, g/m** | Normal | 83.1 | 82.8 | 89.5 | 83.4 | **0.0022** |
|  | Overweight | 91.8 | 95.1 | 96.9 | 101.0 |  |
|  | Obese | 100.0 | 114.2 | 106.8 | 111.5 |  |
| **LVMI-height^2.7, g/m** | Normal | 34.0 | 33.3 | 36.9 | 34.3 | **0.0031** |
|  | Overweight | 37.6 | 39.1 | 40.0 | 41.8 |  |
|  | Obese | 41.2 | 46.6 | 44.1 | 46.1 |  |
| **LVMI-BSA, g/m^2^** | Normal | 81.3 | 80.1 | 86.7 | 81.1 | **0.0347** |
|  | Overweight | 81.3 | 84.0 | 85.5 | 88.5 |  |
|  | Obese | 80.0 | 87.9 | 84.5 | 86.8 |  |
| **LVWT, cm** | Normal | 1.73 | 1.71 | 1.83 | 1.78 | **0.0017** |
|  | Overweight | 1.81 | 1.89 | 1.88 | 1.93 |  |
|  | Obese | 1.89 | 2.03 | 1.97 | 2.03 |  |
| **RWT** | Normal | 0.36 | 0.35 | 0.38 | 0.38 | **0.04** |
|  | Overweight | 0.37 | 0.39 | 0.38 | 0.39 |  |
|  | Obese | 0.38 | 0.40 | 0.39 | 0.41 |  |
| **MAPSE, cm** | Normal | 1.57 | 1.54 | 1.53 | 1.52 | 0.81 |
|  | Overweight | 1.58 | 1.51 | 1.56 | 1.53 |  |
|  | Obese | 1.60 | 1.57 | 1.57 | 1.55 |  |
| **GCS (%)** | Normal | -29.6 | -28.1 | -30.3 | -30.2 | 0.13 |
|  | Overweight | -29.6 | -29.4 | -30.6 | -28.1 |  |
|  | Obese | -29.6 | -27.6 | -30.1 | -26.6 |  |

Least squares means of left ventricular mass index (LVMI) indexed by height, height^2,7, and body surface area, left ventricular wall thickness (LVWT), relative wall thickness (RWT), mitral annular plane systolic excursion (MAPSE), and global circumferential strain (GCS) according to body mass index (BMI) category (normal weight: BMI < 25kg/m^2^, overweight: 25kg/m^2^ ≤ BMI < 30kg/m^2^, obese: BMI ≥ 30kg/m^2^), hypertension status (HTN), and diabetes status (DM). All models are adjusted for cohort, age, sex.

P-Value <0.05 was considered significant for interaction terms.
