# Supplementary material for: Joint influences of obesity, diabetes, and hypertension on indices of ventricular remodeling: Findings from the community-based Framingham Heart Study
Source: PLoS One. 2020 Dec 10;15(12):e0243199. doi: 10.1371/journal.pone.0243199 (PMC7728232; doi:10.1371/journal.pone.0243199)
Supplement: S3 Table — Frequencies (percentage) of LV geometry category by BMI category (normal weight: BMI < 25kg/m2, overweight: 25kg/m2 ≤ BMI < 30kg/m2, obese: BMI ≥ 30kg/m2), hypertension status (HTN) and diabetes status (DM). Percentages represent frequency/row total. (DOCX) [file pone.0243199.s005.docx]

**S3 Table.** Frequencies of left ventricular geometry category by BMI category, hypertension status and diabetes status

|  | Normal left ventricular geometry  N= 4449 | Concentric remodeling  N=767 | Concentric hypertrophy  N=191 | Eccentric hypertrophy  N=334 |
| --- | --- | --- | --- | --- |
| Normal weight, no HTN, no DM, n (%), (N=1838) | 1637 (89.1) | 104 (5.7) | 8 (0.4) | 89 (4.8) |
| Normal weight, HTN, no DM, n (%), (N=346) | 202 (58.4) | 71 (20.5) | 29 (8.4) | 44 (12.7) |
| Normal weight, no HTN, DM, n (%), (N=17) | 13 (76.5) | 1 (5.9) | 0 (0.0) | 3 (17.6) |
| Normal weight, HTN, DM, n (%), (N=28) | 14 (50.0) | 9 (32.1) | 1 (3.6) | 4 (14.3) |
| Overweight, no HTN, no DM, n (%), (N=1436) | 1214 (84.5) | 157 (10.9) | 18 (1.3) | 47 (3.3) |
| Overweight, HTN, no DM, n (%), (N=645) | 408 (63.3) | 128 (19.8) | 49 (7.6) | 60 (9.3) |
| Overweight, DM, no HTN, n (%), (N=30) | 18 (60.0) | 7 (23.3) | 2 (6.7) | 3 (10.0) |
| Overweight, HTN, DM n (%), (N=75) | 34 (45.3) | 17 (22.7) | 11 (14.7) | 13 (17.3) |
| Obese, no HTN, no DM, n (%), (N=662) | 518 (78.2) | 108 (16.3) | 17 (2.6) | 19 (2.9) |
| Obese, HTN no DM, n (%), (N=505) | 312 (61.8) | 114 (22.6) | 39 (7.7) | 40 (7.9) |
| Obese, DM, no HTN, n (%), (N=38) | 21 (55.3) | 12 (31.6) | 3 (7.9) | 2 (5.3) |
| Obese, HTN, DM, n (%), (N=121) | 58 (47.9) | 39 (32.2) | 14 (11.6) | 10 (8.3) |

Frequencies (percentage) of LV geometry category by BMI category (normal weight: BMI < 25kg/m^2^, overweight: 25kg/m^2^ ≤ BMI < 30kg/m^2^, obese: BMI ≥ 30kg/m^2^), hypertension status (HTN) and diabetes status (DM). Percentages represent frequency/row total
