# Supplementary material for: Joint influences of obesity, diabetes, and hypertension on indices of ventricular remodeling: Findings from the community-based Framingham Heart Study
Source: PLoS One. 2020 Dec 10;15(12):e0243199. doi: 10.1371/journal.pone.0243199 (PMC7728232; doi:10.1371/journal.pone.0243199)
Supplement: Attachment — Submitted filename: Response to Reviewers_Xanthakis.docx [file pone.0243199.s006.docx]

Response to Reviewers for manuscript ID# PONE-D-20-14346

Dear Editors and Reviewers,

Thank you for the thoughtful review of our manuscript “**Joint influences of obesity, diabetes, and hypertension on indices of ventricular remodeling: findings from the community-based Framingham Heart Study”.** We have revised the manuscript to meet the journal requirements as well as to address each of the comments of the reviewers. We hope the revised manuscript is suitable for publication in *PLOS ONE*.

Journal Requirements: When submitting your revision, we need you to address these additional requirements.

1. Please ensure that your manuscript meets PLOS ONE's style requirements, including those for file naming.

Response: We have changed the file naming of the supporting information so that it now meets PLOS ONE’s style requirements. We have also changed the subheadings within the abstract so that they are now written in sentence case.

2. Our internal editors have evaluated your manuscript and determined that it is within the scope of our 'Primary and Secondary Prevention of Cardiovascular Disease' Call for Papers. This collection of papers is headed by a team of Guest Editors for PLOS ONE and will encompass a diverse range of research articles.

Additional information can be found on our announcement page: (https://collections.plos.org/s/prevention-cardiovascular).

If you would like your manuscript to be considered for this collection, please let us know in your cover letter and we will ensure that your paper is treated as if you were responding to this call. If you would prefer to remove your manuscript from collection consideration, please specify this in the cover letter.

Response: We thank the editors for their kind offer to include our manuscript in the ‘Primary and Secondary Prevention of Cardiovascular Disease’ Call for papers. After careful consideration we have decided to remove our manuscript from the collection consideration and have stated this in the cover letter.

3. Thank you for stating the following in the Financial Disclosure section:

'This work was partially supported by the National Heart, Lung and Blood Institute’s Framingham Heart Study (Contracts N01-HC-25195, HHSN268201500001I and 75N92019D00031) and by grants HL076784, G028321, HL070100, HL060040, HL080124, HL071039, HL077447, HL107385, HL126136, 2R01HL092577, 1R01HL128914, 1P50HL120163, R01HL131532, R01HL134168, R01HL143227, and 2-K24-HL04334. DDM was supported by grants U54HL143541, R01HL126911, R01HL137734, R01HL137794, R01HL135219, and R01HL141434 from the National Heart, Lung and Blood Institute and National Center for Complementary and Integrative Health (National Institute of Health), and Grant NSF-12-512 from the National Science Foundation. BVJ was supported by the German Heart Foundation / German Foundation of Heart Research. Dr. Vasan is supported by an Evans Scholar award and Jay and Louis Coffman Foundation from the Department of Medicine, Boston University School of Medicine.'

We note that one or more of the authors are employed by a commercial company: Cardiovascular Engineering, Inc

a. Please provide an amended Funding Statement declaring this commercial affiliation, as well as a statement regarding the Role of Funders in your study. If the funding organization did not play a role in the study design, data collection and analysis, decision to publish, or preparation of the manuscript and only provided financial support in the form of authors' salaries and/or research materials, please review your statements relating to the author contributions, and ensure you have specifically and accurately indicated the role(s) that these authors had in your study. You can update author roles in the Author Contributions section of the online submission form.

Please also include the following statement within your amended Funding Statement.

“The funder provided support in the form of salaries for authors [insert relevant initials], but did not have any additional role in the study design, data collection and analysis, decision to publish, or preparation of the manuscript. The specific roles of these authors are articulated in the ‘author contributions’ section.”

If your commercial affiliation did play a role in your study, please state and explain this role within your updated Funding Statement.

Response: The section in the manuscript file reads as follows (page 32, lines 12-23):

Gary F. Mitchell was supported by Cardiovascular Engineering., Inc; this funder provided support in the form of salary for Gary F. Mitchell and various employees of Cardiovascular Engineering, Inc., who are involved in analysis of hemodynamic data. The aforementioned salary support is derived from the various NIH grants that supported the study. Cardiovascular Engineering, Inc., did not have any additional role in the study design, data collection and analysis, decision to publish, or preparation of the manuscript outside of the work done through these NIH grants. The specific role of this author is articulated in the ‘author contributions’ section. There was no additional external funding received for this study.

We now mention the same in the cover letter.

b. Please also provide an updated Competing Interests Statement declaring this commercial affiliation along with any other relevant declarations relating to employment, consultancy, patents, products in development, or marketed products, etc.

Within your Competing Interests Statement, please confirm that this commercial affiliation does not alter your adherence to all PLOS ONE policies on sharing data and materials by including the following statement: "This does not alter our adherence to PLOS ONE policies on sharing data and materials.” (as detailed online in our guide for authors http://journals.plos.org/plosone/s/competing-interests) . If this adherence statement is not accurate and there are restrictions on sharing of data and/or materials, please state these. Please note that we cannot proceed with consideration of your article until this information has been declared.

Response: We now list this information in the manuscript (page 33, lines 3-8) and in the cover letter:

Susan Cheng has received consulting fees from Zogenix for work unrelated to this manuscript.

Gary F. Mitchell has the following disclosures: a) grants: NIH, Novartis (both significant); b) consulting: Novartis, Servier, Merck, Bayer (all significant); and c) ownership: Cardiovascular Engineering, Inc. (significant). These affiliations do not alter our adherence to PLOS ONE policies on sharing data and materials.

We have also changed the heading to “Competing interest statement”.

c. Please include both an updated Funding Statement and Competing Interests Statement in your cover letter. We will change the online submission form on your behalf.

Response: We have now included both updated statements in our cover letter.

Please know it is PLOS ONE policy for corresponding authors to declare, on behalf of all authors, all potential competing interests for the purposes of transparency. PLOS defines a competing interest as anything that interferes with, or could reasonably be perceived as interfering with, the full and objective presentation, peer review, editorial decision-making, or publication of research or non-research articles submitted to one of the journals. Competing interests can be financial or non-financial, professional, or personal. Competing interests can arise in relationship to an organization or another person. Please follow this link to our website for more details on competing interests: http://journals.plos.org/plosone/s/competing-interests

Reviewers' comments:

Reviewer's Responses to Questions

Comments to the Author

1. Is the manuscript technically sound, and do the data support the conclusions?

The manuscript must describe a technically sound piece of scientific research with data that supports the conclusions. Experiments must have been conducted rigorously, with appropriate controls, replication, and sample sizes. The conclusions must be drawn appropriately based on the data presented.

Reviewer #1: Yes

Reviewer #2: Yes

2. Has the statistical analysis been performed appropriately and rigorously?

Reviewer #1: I Don't Know

Reviewer #2: Yes

3. Have the authors made all data underlying the findings in their manuscript fully available?

The PLOS Data policy requires authors to make all data underlying the findings described in their manuscript fully available without restriction, with rare exception (please refer to the Data Availability Statement in the manuscript PDF file). The data should be provided as part of the manuscript or its supporting information, or deposited to a public repository. For example, in addition to summary statistics, the data points behind means, medians and variance measures should be available. If there are restrictions on publicly sharing data—e.g. participant privacy or use of data from a third party—those must be specified.

Reviewer #1: Yes

Reviewer #2: Yes

4. Is the manuscript presented in an intelligible fashion and written in standard English?

PLOS ONE does not copyedit accepted manuscripts, so the language in submitted articles must be clear, correct, and unambiguous. Any typographical or grammatical errors should be corrected at revision, so please note any specific errors here.

Reviewer #1: Yes

Reviewer #2: Yes

5. Review Comments to the Author

Please use the space provided to explain your answers to the questions above. You may also include additional comments for the author, including concerns about dual publication, research ethics, or publication ethics. (Please upload your review as an attachment if it exceeds 20,000 characters)

Reviewer #1: Von Jeinsen et al. utilized data from the Framingham study to investigate the impact of obesity, hypertension and diabetes on cardiac remodeling. English language is fine, but some punctuation typos are present. The paper is well written and the topic interesting.

I suggest providing an image (maybe more than one panel) depicting all echocardiographic measures taken, as not all readers might be familiar with the terms used and a graphical sketch may improve the readability of the manuscript.

Response: We thank the Reviewer for this important suggestion. We have now provided a Figure that details these key measurements, as suggested. The new Figure 1 is now implemented in the manuscript within the methods section. The respective part now reads:

“Measurements of the echocardiographic variables evaluated in this investigation are summarized and depicted in Fig 1.” (page 9, lines 15 and 16)

Fig 1. Schematic presentation of the measurement of the investigated echocardiographic measures.

Panel A shows a 2D parasternal long axis view, lines B and C indicate the position of the m-mode. The respective m-mode view is shown in panels B and C.

Left ventricular end-diastolic dimension (LVDD) and left ventricular end-systolic dimension (LVSD), posterior wall thickness, and septal wall thickness are measured as shown in panel B. Left ventricular mass (LVM), relative wall thickness (RWT) and left ventricular ejection fraction (LVEF) are calculated based on these measures.

Left atrial end-systolic dimension (LASD) and left atrial end-diastolic dimension (LADD) are measured as shown in panel C.

Panel E depicts the basic directions in which circumferential strain (CS) and longitudinal strain (LS) are measured.

Panel F shows the measurements of transmitral Doppler (E and A) and tissue Doppler (e’ and a’) velocities. We have measured early systolic mitral annulus velocity using tissue Doppler imaging at the lateral mitral annulus. E/e’ ratio is calculated based on these measurements.

Mitral annular plane systolic excursion (MAPSE) was measured by the systolic excursion of the mitral annulus from its lowest point at end-diastole (panel G, right) to its highest point at the time of aortic valve closure (panel G, left) at the lateral side of the mitral valve annulus in the apical four-chamber view.

Maximum and minimum volumetric measurements of the LA (LAmax and LAmin) from apical two and four-chamber views were taken at end-diastole for LAmin (panel H, left) and at end-systole for LAmax (panel H, right). Left atrial emptying fraction (LAEF) is calculated based on these measurements.

Reviewer #2: Joint influences of obesity, diabetes, and hypertension on indices of ventricular remodeling: findings from the community-based Framingham Heart Study is a well written study based on 7414 participants in the Offspring and Omni Cohorts of The Framingham Study. The synergistic effect of Hypertension, Obesity and diabetes have earlier been shown for each pair of exposures, but this study adds new knowledge by assessing the joint effect of all three factors on left ventricular structure and function. They add new knowledge by in addition to LVH also assessing left atrial size by systolic diameter, LV longitudinal strain, left atrial emptying fraction and E/e’. They assess the interaction by assessing the association for each parameter in healthy, those with diabetes only, hypertension only and those with both conditions stratified on normal, overweight and obese participants.

They do find an association increasing with increasing number of factors for LVM, LVEF and GLS but not for left atrial diameter or E/e’, the latter both markers of diastolic dysfunction which one would expect to be related to LVM. That GLS is affected stronger than EF is expected as GLS is more sensitive to systolic dysfunction than EF.

Minor comments:

The common abbreviation of early filling velocity over Tissue Doppler early velocity, e prime is usually written E/e’.

Response: We thank Reviewer #2 for pointing this out and we have edited the manuscript accordingly.

Diabetes is often undiagnosed in 20-40% of subjects. Better to use HbA1c >48 mmol/mol (6.5%) to detect these subjects than glucose measurements if HbA1c is available.

Response: We agree that HbA1c is an important measure to detect diabetes. However, HbA1c data were not available at all FHS examinations, therefore we decided to use the present definition that combined values of fasting blood glucose with the use of hypoglycemic medications.

In Table 1 To not describe former smoking often misses clear effects of smoking status.

Response: We thank the Reviewer for this important suggestion and agree that former smoking is important additional information. We, therefore, report the descriptive statistics on the 3-level smoking variable in Table 1 (Page 13). The respective part of Table 1 now reads:

|  | **Overall**  **(n=5741)** | **Normal weight**  **(n=2229)** | **Overweight**  **(n=2186)** | **Obese**  **(n=1326)** |
| --- | --- | --- | --- | --- |
| **Cardiovascular risk factors** | | | | |
| No Smoking, % | 70 | 67 | 71 | 72 |
| Active Smoking, % | 17 | 18 | 17 | 16 |
| Former Smoking, % | 14 | 15 | 12 | 13 |

According to current guidelines indexation is always advised. For LVM use of height¨2.7 is preferred as it has the strongest relation to subsequent outcome (ref ESC guidelines in hypertension ect) As shown in the analyses and earlier shown in the early Framingham studies on LVH indexation by body surface area masks the effect of obesity on LVH. I would therefore drop analyses on LVM/BSA and LVM/h and focus on LVM/h¨^2.7.

Response: We thank the Reviewer for this suggestion and we agree that LVM indexed by height^2.7 is an important measure of LVM. We have therefore performed additional analyses using LVM/hgt^2.7 and present results below as Reviewer Table 1. We have also added these results in the manuscript accordingly (Page 8, line 1; page 12, line 2; page 15, line 8; page 17, line 4; page 18, line 15; and page 25, line 12) and to supplementary tables S1 and S2. We observed similar patterns as those when using LVM indexed by height and LVM indexed by body surface area.

**Reviewer Table 1**. Least square means of *left ventricular mass indexed (LVMI) by height^2.7* by BMI category (A), hypertension status (B), and diabetes status (C) (modeled separately) and jointly (E), adjusted for age, sex, and cohort

| **Trait** | **A) BMI Category** | | | |
| --- | --- | --- | --- | --- |
|  | Normal Weight  (39%) | Over-weight  (38%) | Obese  (23%) | P Value |
| **LVMI indexed by height^2.7** | 34.5 | 38.4 | 42.7 | **<0.0001** |

| **Trait** | **B) Hypertension Status** | | | **C) Diabetes Status** | | |
| --- | --- | --- | --- | --- | --- | --- |
|  | No HTN  (70%) | HTN  (30%) | P Value | No DM  (95%) | DM  (5%) | P Value |
| **LVMI indexed by height^2.7** | 36.6 | 40.7 | **<0.0001** | 37.6 | 41.4 | **<0.0001** |

| **E)** |  |  |  |  |  |  |
| --- | --- | --- | --- | --- | --- | --- |
|  | **BMI Category** | **Healthy** | **DM, no HTN** | **HTN, no DM** | **HTN and DM** | **P-Value for 3-way interaction** |
| **LVMI indexed by height^2.7** | Normal | 34.0 | 33.3 | 36.9 | 34.3 | 0.0031 |
|  | Overweight | 37.6 | 39.1 | 40.0 | 41.8 |  |
|  | Obese | 41.2 | 46.6 | 44.1 | 46.1 |  |

As there was a significant interaction term HT*DMII*BMI, I would drop table 2 and focus on table 3.

Response: We agree that the interaction terms presented in Table 3 play a more central role in our investigation. However, the interaction term was not statistical significant for all variables and we observed interesting patterns e.g. for LVH and arterial hypertension, therefore we believe that the information shown in Table 2 is valuable for the reader. If the Reviewer feels strongly, we would be happy to omit Table 2 or move Table 2 to the supplementary material.

Indexation should also be done for LASd with BSA. See Michael Stylidis Echocardiography. 2019 Mar;36(3):439-450. If LAvolume is available this would be preferred instead of LASd, again indexed by BSA.

Response: We thank the Reviewer for this suggestion and we agree that LA volume would be an interesting parameter for LA measure. Unfortunately, we do not have LA volume measures in our cohort. We performed additional analyses using LASD indexed by BSA (see **Reviewer Table 2** below), but we believe that indexing to BSA might “excuse” for obesity. Using LASD indexed by BSA, we observed smaller values for LASD in obese individuals compared to those with normal weight. We believe that using the LASD values shows a clearer pattern. We hope the Reviewer agrees with our approach.

**Reviewer Table 2**. Least square means of left atrial systolic dimension index by body surface area (BSA) by BMI category (A), hypertension status (B), and diabetes status (C) (modeled separately) and jointly (E), adjusted for age, sex, and cohort

| **Trait** | **A) BMI Category** | | | |
| --- | --- | --- | --- | --- |
|  | Normal Weight  (39%) | Over-weight  (38%) | Obese  (23%) | P Value |
| **LASD, indexed by BSA** | 2.04 | 2.01 | 1.96 | **<0.0001** |

| **Trait** | **B) Hypertension Status** | | | **C) Diabetes Status** | | |
| --- | --- | --- | --- | --- | --- | --- |
|  | No HTN  (70%) | HTN  (30%) | P Value | No DM  (95%) | DM  (5%) | P Value |
| **LASD, indexed by BSA** | 2.00 | 2.02 | **0.0054** | 2.01 | 2.03 | **<0.0001** |

| **E)** |  |  |  |  |  |  |
| --- | --- | --- | --- | --- | --- | --- |
|  | **BMI Category** | **Healthy** | **DM, no HTN** | **HTN, no DM** | **HTN and DM** | **P-Value for 3-way interaction** |
| **LASD, indexed by BSA** | Normal | 2.02 | 2.07 | 1.98 | 2.13 | 0.36 |
|  | Overweight | 1.95 | 1.97 | 1.98 | 2.00 |  |
|  | Obese | 2.00 | 2.03 | 2.02 | 2.11 |  |

It would be appropriate also to mention interobserver data in the echocardiographic measurement.

Response: We agree that information on interobserver data is crucial, therefore we have added this information and the respective references in the methods section as follows:

Page 9, lines 16 and 17: ”‘Data on interobserver correlations have been previously published.(51,53,54)’”

Respective references:

51.	Cheng S, Larson MG, McCabe EL, Osypiuk E, Lehman BT, Stanchev P, et al. Reproducibility of speckle-tracking-based strain measures of left ventricular function in a community-based study. J Am Soc Echocardiogr. 2013;26(11):1258–66.

53.	Sardana M, Nah G, Tsao CW, Ogunsua AA, Vittinghoff E, Thomas RC, et al. Clinical and Echocardiographic Correlates of Left Atrial Function Index: The Framingham Offspring Study. J Am Soc Echocardiogr. 2017;30(9):904-912.e2.

54.	Sundström J, Sullivan L, Selhub J, Benjamin EJ, D’Agostino RB, Jacques PF, et al. Relations of plasma homocysteine to left ventricular structure and function: The Framingham Heart Study. Eur Heart J. 2004;25(6):523–30.

As BMI, LVM, hypertension and diabetes all are correlated, it would strengthen the discussion to also report Variance inflation factor for these associations and a control of residuals to assure the GLM models used to calculate LS means are valid.

Response: We calculated variant inflation factor values for our models, and we did not observe any major collinearity. We have added this information to our manuscript, as follows:

Page 12, lines 6 and 7: “We calculated variance inflation factor values for all models, but we did not observe major multicollinearity.“

The increasing LVEF in subjects with hypertension could be due to concentric hypertrophy leaving the actual LV volume smaller. Then to maintain cardiac output EF has to increase to maintain stroke volume if heart rate does not increase. The finding of a decreased LVEF for obese non hypertensive subjects could be due to the increase in circulating volume seen with obesity thought to be a cause of dilatation and heart failure associated with obesity. This could be tested in this study by assessing LVDd in these groups and see if obese non hypertensive are more dilated than normal non hypertensivs.

Response: We followed the Reviewer’s suggestion and compared LVDd in normal weight/overweight individuals (4.87cm) and obese individuals (5.02cm, p<0.0001) in participants without hypertension. Obese individuals have higher measures of LVDD. However, we believe that the information presented in Figure 2 shows important additional information. Obese individuals without hypertension have higher rates of concentric remodeling (16%) vs. eccentric remodeling (3%). An increase in LVDd is accompanied by an increase in hypertrophy and the decrease in LVEF cannot be explained by dilatation in obese individuals alone. We hope the Reviewer agrees with our approach.

The finding of an increasing MAPSE for increasing BMI is interesting and although not significant the same trend is seen in stratified analysis over all four groups of health, only hypertensive, only diabetes and both. As the decrease in MAPSE over these groups is not significant in overall analysis this is also interesting.

I would suggest that the presentation and discussion focuses on the overall stratification in BMI groups and on healthy, only hypertensive, only diabetes and both and effects disappearing here but apparent in selective two way analysis can be discussed in relation to power or other possible reasons for the lack of overall effect.

Response: We thank the Reviewer for this suggestion. To our knowledge, the increase in MAPSE among overweight and obese individuals compared to normal weight individuals has not been reported before. To address the Reviewer’s comment, we would like to note that we tested for three-way interactions and we did not observe a statistically significant interaction of BMI, HTN, and diabetes with MAPSE, suggesting that there are no synergistic (multiplicative) associations between three conditions and MAPSE. However, additive associations could be present still, and we have tried to describe these associations in the discussion. We agree with the Reviewer that our discussion should be more focused on the observation that MAPSE increased in all four groups (healthy, only hypertensive, only diabetes, and both) with increasing BMI (similar to the trend we observed in 2 way analyses) and have changed the discussion section accordingly, as follows:

Page 26, lines 5-13: “Overweight and obesity were associated with higher (favorable) mean MAPSE values compared to normal weight individuals in all subgroups (i.e., no additional risk factors, only hypertension present, only diabetes present, and with both diabetes and hypertension). Of note, MAPSE was lower (unfavorable) in individuals with hypertension, diabetes, or both if BMI was held constant. Overall these observations are consistent with a possible greater long axis LV shortening (higher MAPSE) with higher BMI category, but attenuated shortening (lower MAPSE) in the presence of concomitant hypertension and diabetes. As mentioned above, additional studies are warranted to confirm this observation and to investigate potential physiological mechanisms.”

6. PLOS authors have the option to publish the peer review history of their article (what does this mean?). If published, this will include your full peer review and any attached files.

If you choose “no”, your identity will remain anonymous but your review may still be made public.

Do you want your identity to be public for this peer review? For information about this choice, including consent withdrawal, please see our Privacy Policy.

Reviewer #1: No

Reviewer #2: Yes: Henrik Schirmer

While revising your submission, please upload your figure files to the Preflight Analysis and Conversion Engine (PACE) digital diagnostic tool, https://pacev2.apexcovantage.com/. PACE helps ensure that figures meet PLOS requirements. To use PACE, you must first register as a user. Registration is free. Then, login and navigate to the UPLOAD tab, where you will find detailed instructions on how to use the tool. If you encounter any issues or have any questions when using PACE, please email PLOS at figures@plos.org. Please note that Supporting Information files do not need this step.

Response: We used PACE to ensure that our figures meet PLOS requirements.
